# Publisher’s Note: A New Addition to the MDPI Portfolio—*Journal of Eye Movement Research*

**DOI:** 10.3390/jemr18010002

**Published:** 2025-01-17

**Authors:** Carla Aloè

**Affiliations:** MDPI AG, Grosspeteranlage 5, 4052 Basel, Switzerland; carla.aloe@mdpi.com

We are delighted to announce that the *Journal of Eye Movement Research* (*JEMR*) has joined the MDPI portfolio. Established in 2007 by Rudolf Groner, *JEMR* has earned its place as a leading journal in the field of ophthalmology, with a focus on the whole spectrum of oculomotor functioning. In recognition of its impact, the journal quickly achieved its first Journal Impact Factor in 2013.

Dr. Groner has served as the Editor-in-Chief since the journal’s founding, guiding it with dedication and expertise. As explained in his editorial, he has now decided to step down from the role [1], and we wish him all the best in his well-earned retirement.

Looking ahead, we are excited to welcome a new Editor-in-Chief to lead *JEMR* into the future, alongside its esteemed editorial board, authors, and readers.

MDPI will take over the publication of *JEMR* from Bern Open Publishing starting with Volume 18 [2], and we look forward to supporting and expanding the journal’s influence in the ophthalmology community. We thank all stakeholders for their trust and collaboration as we embark on this new chapter together.
